# Effects of immunomodulatory drugs on TNF-α and IL-12 production by purified epidermal langerhans cells and peritoneal macrophages

**DOI:** 10.1186/1756-0500-4-24

**Published:** 2011-01-28

**Authors:** Simone R Campelo, Moises B da Silva, José LF Vieira, Jorge P da Silva, Claudio G Salgado

**Affiliations:** 1 Dermato-Immunology Laboratory, Federal University of Pará, Dr Marcello Candia Reference Unit in Sanitary Dermatology of the State of Pará, Marituba, PA, Brazil; 2 Institute of Biological Sciences, Federal University of Pará, Belém, PA, Brazil; 3 Toxicology Laboratory, Federal University of Pará, Belém, PA, Brazil; 4 Faculty of Pharmacy, Federal University of Pará, Belém, PA, Brazil

## Abstract

**Background:**

Langerhans cells constitute a special subset of immature dendritic cells localized in the epidermis that play a key role in the skin's immune response. The production of cytokines is a key event in both the initiation and the regulation of immune responses, and different drugs can be used to remove or modify their production by DC and, therefore, alter immune responses in a broad spectrum of diseases, mainly in human inflammatory and autoimmune diseases. In the present study, we examined the effects of prednisone, thalidomide, cyclosporine A, and amitriptyline, drugs used in a variety of clinical conditions, on the production of TNF-α, IL-10, and IL-12 by purified epidermal Langerhans cells and peritoneal macrophages in BALB/c mice.

**Findings:**

All drugs inhibited TNF-α production by Langerhans cells after 36 hours of treatment at two different concentrations, while prednisone and thalidomide decreased IL-12 secretion significantly, amitriptyline caused a less pronounced reduction and cyclosporine A had no effect. Additionally, TNF-α and IL-12 production by macrophages decreased, but IL-10 levels were unchanged after all treatments.

**Conclusions:**

Our results demonstrate that these drugs modulate the immune response by regulating pro-inflammatory cytokine production by purified epidermal Langerhans cells and peritoneal macrophages, indicating that these cells are important targets for immunosuppression in various clinical settings.

## Background

Dendritic cells (DC) are professional antigen-presenting cells (APC) that possess the unique ability to stimulate naïve T cells and initiate a primary immune response [1]. In the skin, the main DC populations present include epidermal DC (Langerhans cells) and dermal DC (myeloid DC and plasmacytoid DC). Langerhans cells (LC) are immature cells that reside in the epidermal layer and are distinct from other DC subsets [2].

In medicine, LC are often studied due to their role in numerous skin diseases, including psoriasis and contact and allergic dermatitis [3], and their ability to uptake antigen is crucial to inducing dermal immune response and tolerance [4]. Upon activation, LC gain the ability to produce chemokines [5] and pro-inflammatory cytokines, including tumor necrosis factor-α (TNF-α) and IL-12 [6], which coordinate local and systemic inflammatory responses. TNF- α is a pleiotropic cytokine, produced primarily by monocytes and macrophages, which plays an important role in host immune responses. Antigen-presenting cells and phagocytic cells, including monocytes and macrophages, dendritic cells, and neutrophils, also are the primary producers of IL-12, an important regulatory cytokine that has a function central to the initiation and regulation of the adaptive immune response [7]. IL-10 is also an important immunoregulatory cytokine produced by many cell populations. Its main biological function seems to be the limitation and termination of inflammatory responses and the regulation of differentiation and proliferation of several immune cells, and the major source of IL-10 in vivo seems to be macrophages [8].

Different drugs may be used to modify cytokine production by DC and thus alter the initiation and regulation of immune responses to a broad spectrum of diseases, such as human inflammatory and autoimmune diseases [9]. Immunosuppressive drugs used to treat dermatological conditions, control allograft rejection, and promote transplant tolerance are well recognized for their ability to inhibit lymphocyte activation and proliferation. These drugs may also affect the differentiation, viability, and functions of DC [10], resulting in suppressed T-cell responses. Such drugs promote T-cell unresponsiveness as a means for treating a variety of clinical conditions, including transplantation and autoimmune disorders and allergic hypersensitivity.

LC and macrophages (MΦ) are effective APC whose secretion of immunoregulatory and pro-inflammatory cytokines plays a critical role during T-cell priming [6]. To gain a better understanding of immunosuppressive drugs' influences on these APC and their potential to induce tolerance, the present study sought to examine the effects of prednisone, thalidomide, cyclosporine A, and amitriptyline on TNF-α, IL-10, and IL-12 production by epidermal LC and peritoneal MΦ *in vitro*.

## Methods

### Reagents

Prednisone, thalidomide, cyclosporine A, amitriptyline and LPS were purchased from Sigma-Aldrich (St. Louis, MO) and were dissolved in dimethyl sulfoxide (DMSO) or methanol to make 10^-2 ^M stock solutions. ELISA kits for TNF-α, IL-12 (p40/p70), and IL-10 were purchased from BD Pharmingen (San Diego, CA).

### Mice

Female BALB/c mice were provided by the Evandro Chagas Institute, where they were maintained under specific pathogen-free conditions until use at the age of 8-12 weeks. All procedures were carried out under the Brazilian Law 1153-A, which regulates animal research in Brazil, and were approved by animal ethics committee of Pará Federal University.

### LC enrichment and culture

LC were prepared using the previously described panning method, resulting in a purity of over 95% [11]. Briefly, the murine epidermis was separated from the dermis after 3 h of treatment with dispase II (3000 U per ml, Sigma), a neutral protease, at 37°C and 5% CO_2_. The epidermis was then incubated with DNAse enzyme (0.025%, Sigma) for 20 min at room temperature, after which an epidermal cell suspension was obtained by vigorous pipetting of the epidermal sheets. Next, the cell suspension was treated with mouse anti-mouse Ia^d ^(murine MHC allele) monoclonal antibody (1:600, BD PharMingen, San Diego, CA) for 45 min on ice. The cells were then incubated in plates coated with goat anti-mouse IgG (Jackson Immuno Research, West Grove, PA) (1:100) for an additional 45 min at 4°C. After washing away floating cells, adherent LC were collected and resuspended in complete medium, consisting of RPMI-1640 supplemented with 10% fetal calf serum (Gibco, Grand Island, NY), 10000 U/ml penicillin/streptomycin solution (Sigma), and 50 μM β-mercaptoethanol (Merck, Darmstadt, Germany), dispensed into 96-well flat bottom plates and incubated in humidified 5% CO_2 _at 37°C. These cells are cultured in suspension and can be maintained in culture flasks that are not tissue-culture treated.

### Cell viability assessment

After exposure to prednisone, thalidomide, cyclosporine A, or amitriptyline for 36 h, LC or MΦ were rinsed three times in phosphate-buffered saline (PBS) and incubated with propidium iodide (10 μg/ml) immediately prior to flow cytometric analysis (Epics XL, Beckman Coulter, Miami, FL) or before mounting in dilute medium on a glass slide with coverslip to assess cell viability.

### Preparation of peritoneal MΦ

MΦ were isolated from the peritoneal cavities of female BALB/c mice. Briefly, 10 ml of cold PBS was injected into the peritoneal cavity of each mouse and the resultant exudate was immediately collected, washed, and resuspended in RPMI-1640 medium supplemented with 10% fetal calf serum, penicillin/streptomycin solution and 50 μM β-mercaptoethanol. The cell suspension was then dispensed into 24-well flat bottom plates and incubated in humidified 5% CO_2 _at 37°C for 1 h to allow MΦ adherence. The non-adherent cells were removed by three washes with RPMI-1640 medium. The purified MΦ were incubated for an additional 24 h with 1 ml RPMI-1640 medium supplemented with 10% fetal calf serum, penicillin/streptomycin solution and 50 μM β-mercaptoethanol and 10 ng/ml lipopolysaccharide (LPS; Sigma) [12].

### Drug treatment

In the culture experiments, purified LC (2 × 10^5 ^cells/well) or peritoneal MΦ (5 × 10^4 ^cells/well) were incubated with or without prednisone, thalidomide, cyclosporine A, or amitriptyline at varying concentrations (10^-6 ^M, 10^-8 ^M, or diluent alone) in RPMI-1640 medium supplemented with 10% fetal calf serum, 10000 U/ml penicillin/streptomycin solution and 50 μM β-mercaptoethanol. Drug concentrations were chosen based on the results of preliminary studies which showed that these concentrations had no effects on the functions of other cell types [13,14].

### Measurement on cytokine production

Culture supernatants were collected after 36 h, centrifuged, stored at -20°C and subjected to protein quantification at the indicated time-points by ELISA, using mouse TNF-α, IL-12, and IL-10 immunoassay kits according to the manufacturer's instructions (BD Pharmingen). Protein levels were assessed using a microplate reader at 450 nm (MRX Revelation-DINEX, Chantilly, VA), and each sample was tested in triplicate. Data are expressed in pg/ml × 10^5 ^cells (LC) or pg/ml × 10^4 ^cells (peritoneal MΦ).

### Statistical analysis

Data obtained from three independent experiments are presented as mean ± SD and were compared using the Student's t test for single comparisons or analysis of variance (ANOVA) for multiple comparisons. Differences were considered significant at p < 0.05.

## Results

### LC viability by propidium iodide staining

The viability of cultured LC was carefully checked in each experiment. By panning, we obtained about 95% of LC, with more than 90% viability (Table 1). After 36 hours in culture, cell viability decreased to approximately 70%, and was not affected by treatment with prednisone, thalidomide, cyclosporine A, or amitriptyline at 10^-6 ^M or 10^-8 ^M (Table 1).

**Table 1:** LC viability after treatment with immunomodulatory drugs^a^

	Concentration	Viability (%)
Freshly isolated LC	-	94.4 ± 2.1
36 h cultured LC	-	73.3 ± 2.8
Prednisone	10^-6 ^M	69.9 ± 3.7
	10^-8 ^M	66.7 ± 5.5
Thalidomide	10^-6 ^M	63.5 ± 3.2
	10^-8 ^M	61.4 ± 2.1
Cyclosporine A	10^-6 ^M	62.2 ± 6.4
	10^-8 ^M	61.2 ± 10.7
Amitriptyline	10^-6 ^M	66.2 ± 2.2
	10^-8 ^M	64.6 ± 1.7

^a^Purified LC were cultured for 36 h in the presence or absence of 10^-6 ^M or 10^-8 ^M prednisone, thalidomide, cyclosporine A, or amitriptyline. Cell viability was then assessed using propidium iodide. All results are shown as mean ± SD for three independent experiments, which did not vary significantly from the control.

### Differential effect of immunomodulatory drugs on cytokine secretion by LC

The purified LC acquires mature phenotypes during culture even without exogenous stimulation [15]. We utilized those cells for *in vitro *culture system in which the interference of keratinocytes or keratinocytes-derived cytokines was negligible. To determine whether TNF-α and IL-12 were secreted into the culture medium by unstimulated LC after 36 hours, cytokine levels in the medium were quantified via enzyme-linked immunosorbent assay (ELISA). TNF-α production (21.8 ± 1.4 pg/ml × 10^5 ^cells) decreased to 10.8 pg/ml × 10^5 ^cells (p < 0.05) after treatment with 10^-6 ^M prednisone and to 9.5 pg/ml × 10^5 ^cells (p < 0.05) after treatment with 10^-8 ^M prednisone, corresponding to an almost 50% reduction (Table 2). LC treatment with thalidomide resulted in significant inhibition of TNF-α secretion, decreasing from 21.8 ± 1.4 pg/ml × 10^5 ^cells to 7.8 ± 1.7 pg/ml × 10^5 ^cells at 10^-6 ^M (64% reduction, p < 0.05) and to 4.4 ± 3.8 pg/ml × 10^5 ^cells at 10^-8 ^M (80% reduction, p < 0.01) (Table 2). Following cyclosporine A treatment, TNF-α production was lowered to 5.8 ± 0.4 pg/ml × 10^5 ^cells at 10^-6 ^M (73% reduction, p < 0.01) and to 7.6 ± 0.5 pg/ml × 10^5 ^cells at 10^-8 ^M (65% reduction, p < 0.01) (Table 2). Similarly, TNF-α release by LC was reduced by amitriptyline, but this reduction was less pronounced than that induced by each of the three other compounds over the same time period. Specifically, amitriptyline decreased TNF-α secretion by 55% at 10^-6 ^M (9.7 ± 2.2 pg/ml × 10^5 ^cells, p < 0.05) and by 44% at 10^-8 ^M (12.2 ± 0.6 pg/ml × 10^5 ^cells, p < 0.05). IL-12 production (9.4 ± 0.5 pg/ml × 10^5 ^cells) decreased to 2.9 ± 0.7 pg/ml × 10^5 ^cells (p < 0.01) after treatment with 10^-6 ^M prednisone and to 4.0 ± 1.1 pg/ml × 10^5 ^cells (p < 0.01) after treatment with 10^-8 ^M prednisone, corresponding to a 69% and 57% reduction, respectively (Table 2). LC treatment with thalidomide resulted in significant inhibition of IL-12 production, decreasing from 9.4 ± 0.5 pg/ml × 10^5 ^cells to 4.8 ± 0.5 pg/ml × 10^5 ^cells at 10^-6 ^M (49% reduction, p < 0.01) and to 3.5 ± 1.8 pg/ml × 10^5 ^cells at 10^-8 ^M (62% reduction, p < 0.01) (Table 2). Following cyclosporine A treatment, no significant reduction in IL-12 secretion was noted for any of the two concentrations tested (Table 2). Similarly, amitriptyline inhibited IL-12 secretion by 32% at 10^-6 ^M (6.4 ± 0.3 pg/ml × 10^5 ^cells, p < 0.05), while the lowest dose of the same drug did not have a statistically significant effect. Additionally, no IL-10 release was detected in the culture supernatants over the 36-hour incubation period (data not shown).

**Table 2:** *In vitro *effects of two different concentrations of immunomodulatory drugs on TNF-α and IL-12 production by LC^a^

	Concentration	TNF-α (pg/ml)	IL-12 (pg/ml)
36 h cultured LC	-	21.8 ± 1.4	9.4 ± 0.5
Prednisone	10^-6 ^M	10.8 ± 2.0*	2.9 ± 0.7**
	10^-8 ^M	9.5 ± 0.9*	4.0 ± 1.1**
Thalidomide	10^-6 ^M	7.8 ± 1.7*	4.8 ± 0.5**
	10^-8 ^M	4.4 ± 3.8**	3.5 ± 1.8**
Cyclosporine A	10^-6 ^M	5.8 ± 0.4**	8.7 ± 0.4
	10^-8 ^M	7.6 ± 0.5**	8.5 ± 0.6
Amitriptyline	10^-6 ^M	9.7 ± 2.2*	6.4 ± 0.3*
	10^-8 ^M	12.2 ± 0.6*	7.4 ± 2.7

^a^Purified LC were cultured for 36 h in the presence or absence of 10^-6 ^M or 10^-8 ^M prednisone, thalidomide, cyclosporine A, or amitriptyline. TNF-α and IL-12 levels in the culture supernatant were quantified by ELISA. All results are shown as mean ± SD for three independent experiments. *p < 0.05 vs. control. **p < 0.01 vs. control.

### MΦ viability by propidium iodide staining

After exposure to prednisone, thalidomide, cyclosporine A, or amitriptyline for 36 h, we assessed the cell viability of cultured MΦ by PI staining. None of the immunomodulatory drugs used affected the cell viability of cultured MΦ (Table 3).

**Table 3:** Peritoneal MΦ viability after treatment with immunomodulatory drugs^a^

	Concentration	Viability (%)
36 h cultured Mϕ	-	95.8 ± 1.5
Prednisone	10^-6 ^M	84.2 ± 2.5
	10^-8 ^M	86.1 ± 1.7
Thalidomide	10^-6 ^M	85.4 ± 3.2
	10^-8 ^M	86.9 ± 2.2
Cyclosporine A	10^-6 ^M	85.5 ± 2.3
	10^-8 ^M	88.2 ± 3.7
Amitriptyline	10^-6 ^M	83.8 ± 2.9
	10^-8 ^M	87.7 ± 0.8

^a^Purified MΦ were first pre-incubated for 24 h with 10 ng/ml LPS, and then cultured for 36 h in the presence or absence of 10^-6 ^M or 10^-8 ^M prednisone, thalidomide, cyclosporine A, or amitriptyline. Cell viability was then assessed using propidium iodide. All results are shown as mean ± SD for three independent experiments, which did not vary significantly from the control.

### Differential effect of immunomodulatory drugs on cytokine secretion by MΦ

MΦ incubated with LPS for 36 hours secreted TNF-α, IL-12 and IL-10 at the following respective levels: 294 ± 33.9 pg/ml × 10^4 ^cells, 258 ± 27.7 pg/ml × 10^4 ^cells, and 195 ± 12.8 pg/ml × 10^4 ^cells (Table 4). A significant decrease in TNF-α production by LPS-stimulated MΦ was observed after prednisone treatment at 10^-6 ^M or 10^-8 ^M (p < 0.05), corresponding with a 56% or 53% (129.6 ± 33.0 pg/ml × 10^4 ^cells or 138.2 ± 6.0 pg/ml × 10^4 ^cells) reduction. Thalidomide at 10^-6 ^M downregulated TNF-α secretion by 65.6%, to 100.9 ± 9.0 pg/ml × 10^4 ^cells (p < 0.01), while LPS-stimulated MΦ incubated with 10^-8 ^M thalidomide exhibited a slight, but statistically insignificant, reduction in TNF-α release. Similarly, cyclosporine A reduced TNF-α secretion significantly (117.4 ± 59.7 pg/ml × 10^4 ^cells) but only at the highest dose, while the lowest dose of the same drug did not have a statistically significant effect. When LPS-stimulated MΦ were incubated with 10^-6 ^M amitriptyline, TNF-α release was reduced from 339.3 ± 82.3 pg/ml × 10^4 ^cells to 120.6 ± 16.9 pg/ml × 10^4 ^cells (60% reduction, p < 0.05), while 10^-8 ^M amitriptyline caused no significant reduction (Table 4).

**Table 4:** *In vitro *effects of two different concentrations of immunomodulatory drugs on TNF-α, IL-12, and IL-10 production by MΦ^a^

	Concentration	TNF-α (pg/ml)	IL-12 (pg/ml)	IL-10 (pg/ml)
36 h cultured Mϕ	-	339.3 ± 82.3	255.1 ± 27.5	195.0 ± 12.8
Prednisone	10^-6 ^M	129.6 ± 33.0*	184.6 ± 48.1	209.8 ± 17.3
	10^-8 ^M	138.2 ± 6.0*	149.4 ± 7.5**	193.5 ± 29.8
Thalidomide	10^-6 ^M	100.9 ± 9.0**	149.0 ± 14.9*	221.2 ± 22.7
	10^-8 ^M	228.6 ± 78.9	192.9 ± 63.1	160.1 ± 29.8
Cyclosporine A	10^-6 ^M	117.4 ± 59.7*	190.6 ± 10.8	212.4 ± 4.8
	10^-8 ^M	215.4 ± 32.3	150.1 ± 11.9**	184.2 ± 21.9
Amitriptyline	10^-6 ^M	139.7 ± 49.2*	157.0 ± 27.6*	200.2 ± 10.2
	10^-8 ^M	198.8 ± 47.2	147.1 ± 47.4*	207.5 ± 8.1

^a^Purified MΦ were pre-incubated for 24 h with 10 ng/ml LPS and cultured for 36 h in the presence or absence of 10^-6 ^M or 10^-8 ^M prednisone, thalidomide, cyclosporine A, or amitriptyline. TNF-α, IL-12, and IL-10 levels in the culture supernatant were quantified by ELISA. All results are shown as mean ± SD for three independent experiments. *p < 0.05 vs. control. **p < 0.01 vs. control.

IL-12 secretion was also significantly downregulated (p < 0.05) by prednisone at 10^-8 ^M (149.4 ± 7.5 pg/ml × 10^4 ^cells), thalidomide at 10^-6 ^M (139.0 ± 9.8 pg/ml × 10^4 ^cells), cyclosporine A at 10^-8 ^M (149.7 ± 9.1 pg/ml × 10^4 ^cells), and amitriptyline at both 10^-6 ^M and 10^-8 ^M (150.0 ± 5.4 pg/ml × 10^4 ^cells and 148.6 ± 8.6 pg/ml × 10^4 ^cells), all corresponding to about a 40% reduction. Meanwhile, IL-10 levels by LPS-stimulated MΦ were not altered by any of the drugs tested (Table 4).

## Discussion

Epidermal DC are believed to be involved in allergic and irritant contact dermatitis [16], as well as in autoimmune disease [17]. One approach to improving DC tolerogenicity is suppression of their maturation using anti-inflammatory cytokines or pharmacological agents [18]. In the present study, we demonstrate that several immunomodulatory drugs markedly downregulated TNF-α and IL-12 secretion by unstimulated cultured purified LC and by LPS-stimulated MΦ without diminishing cell viability.

We found that *in vitro *TNF-α and IL-12 production by unstimulated cultured LC was reduced by prednisone. Previous data demonstrated that DC derived from human monocytes were similarly suppressed by dexamethasone [19]. Our results show that prednisone also inhibits LPS-stimulated MΦ production of TNF-α, corroborating a previous study that demonstrated suppressed TNF-α secretion by peripheral blood monocytes pre-incubated with LPS for 24 or 48 hours, and then treated with dexamethasone. The same study also showed that, depending on the amplitude of LPS stimulation, glucocorticoids increased IL-10 secretion at low doses and decreased IL-10 release at high doses [20]. Other research showed that methylprednisolone consistently induces IL-10 production by human alveolar MΦ when cells are exposed to the drug for up to 20 hours, followed by LPS stimulation [21]. All of the *in vitro *data summarized above appear to disagree with our findings, since we did not see a consistent change in IL-10 production due to prednisone treatment. This discrepancy may be due to differences in the experimental setup, including the fact that previous studies added glucocorticoids before or together with LPS and used human cells. Thus, the type of stimulus and cell source may influence the effects of glucocorticoids on IL-10 secretion.

Prednisone also inhibited the IL-12 secretion by LPS-stimulated MΦ after 36 hours of treatment. This finding corroborates published data showing that MΦ treated with dexamethasone for 18 hours, followed by stimulation with Listeria antigen for two days, exhibited significantly reduced IL-12 production [22].

Thalidomide has been shown to profoundly inhibit the ability of LC to present skin-purified antigens and to produce TNF-α [23]. Our data were consistent with these earlier findings, demonstrating that thalidomide markedly reduced TNF-α generation by unstimulated cultured LC after 36 hours of treatment. Previous studies also demonstrated that thalidomide has inhibitory effects on TNF-α secretion by unstimulated peripheral blood cells treated for two days [13]. Thalidomide may curtail TNF-α production by inhibiting degradation of the inhibitor of kappa B (IκB) and thus, NF-κB-mediated expression of TNF-α mRNA [24]. Here, we also detected downregulation of TNF-α production by LPS-stimulated MΦ after thalidomide treatment. Furthermore, we found also that IL-12 production by unstimulated cultured LC was strongly suppressed by both concentrations of thalidomide, and that IL-12 secretion by LPS-stimulated MΦ was reduced by high concentrations of thalidomide (10^-6 ^M), supporting previous studies showing that thalidomide inhibits IL-12 production by LPS-stimulated monocytes [25]. Another study suggested suppression of TNF-α and IL-12 as a possible mechanism of thalidomide's clinical effects in Crohn's disease, which improves clinical symptoms in patients [26], what may explain its clinical efficacy.

We next examined the effects of cyclosporine A on unstimulated cultured LC. TNF-α production by LC was inhibited at both concentrations of cyclosporine A, suggesting that the effects of this drug are similar to those of prednisone and thalidomide. However, we observed no significant changes in the IL-12 secretion by cyclosporine A-treated LC, despite previous observations that the drug blocked IL-12 production by CD40-stimulated monocyte-derived DC [19]. It is possible that cyclosporine A exerts inhibitory effects at different sites in these two types of APC, resulting in inhibited IL-12 secretion in DC but not in LC. This divergent outcome may be due to the cells' different maturation states or levels of IL-12 production [27].

Although the mechanism underlying cyclosporine A effects on LC remains to be elucidated, our results support the hypothesis that cyclosporine A inhibits unstimulated cultured LC TNF-α secretion.

Other investigators have also observed decreased basal TNF-α secretion by the monocyte cell line U936 [28] cultured with cyclosporine A for 18 hours at various concentrations, in either the presence or the absence of LPS. Another study has demonstrated that cyclosporine A inhibits IL-12 production and stimulates IL-10 secretion by subtypes of peripheral blood DC (CD11c+ and CD11c-) [29]. In our study, we observed that cyclosporine A inhibition of TNF-α and IL-12 production by LPS-stimulated MΦ only occurred at the highest drug concentration. Recently, it was reported that the immunomodulatory effects of cyclosporine A may be dose-dependent and may be due to inhibition of such transcription factors such as NF-κB and activator protein-1 (AP-1) by regulating the Ca+ signaling pathway (calmodulin and calmodulin-dependent protein kinase-II, or CaMK-II) [30]. Although the effects of cyclosporine A on LC cytokine production are not well understood, the drug may act by suppressing the number, DNA synthesis, and function of these cells [31].

We also examined the effects of amitriptyline on LC and MΦ cytokine secretion. Despite recent work showing that amitriptyline plays an immunomodulatory role, little is known about its mechanism of action and target immune cells. It was previously reported that similar tricyclic antidepressants, such as clomipramine, imipramine, and citalopram, cause reduction in TNF-α release by LPS-stimulated peripheral blood monocytes [32]. Recently, one study demonstrated that amitriptyline and its metabolite, nortriptyline, decreases TNF-α secretion by glial cells [33], which take part in the immune response of central nervous system. However, other recent research using whole blood stimulated by LPS or concanavalin A did not detect any effect of such antidepressants as desipramine, clomipramine, and trimipramine on TNF-α and IL-12 production [34]. Meanwhile, our data revealed that amitriptyline inhibits TNF-α and IL-12 secretion by both cell types studied, confirming previous studies using cultured cells. While the immunomodulatory activity of antidepressants on cytokine production is not yet fully characterized, it is believed that one underlying mechanism is an increase in intracellular cyclic adenosine monophosphate (cAMP) [32]. These drugs may also influence immunocompetent cells cytokine secretion by binding to surface serotonin receptors [35]. Some researchers may not have observed similar antidepressant effects on immunocompetent cells TNF-α secretion because they did not specifically use non-tricyclic antidepressants. Moreover, many of these studies analyzed whole blood, which may contain other cells that affect TNF-α production by releasing cytokines or even by direct cell-cell contact.

In summary, the study indicates that there are differential regulation by immunosuppressive drugs on TNF-α and IL-12 production by LC and MΦ, which constitute important targets for immunomodulatory drugs. Further *in vitro *and *in vivo *studies are necessary to substantiate these findings and to provide further information on the mode of action of prednisone, thalidomide, cyclosporine A and amitriptyline on a cellular and molecular level.

## List of abbreviations

APC: antigen-presenting cells; DC: dendritic cells; ELISA: enzyme-linked immunosorbent assay; IL-10: interleukin-10; IL-12: interleukin-12; LC: Langerhans cells; LPS: lipopolysaccharide; MΦ: macrophages; NF-κB: nuclear factor-kappa B; TNF-α: tumor necrosis factor-α.

## Competing interests

The authors declare that they have no competing interests.

## Authors' contributions

SRC participated in the design of the study, carried out some of the literature searches, conducted the experiments and drafted the manuscript. MBS participated in the design of the study, conducted the experiments and synthesized the findings. JLFV provided some drugs for the study. JPS participated in the design of the study and carried out some of the literature searches. CGS participated in the design of the study, synthesized the findings and edited the manuscript. All the authors read and approved the final manuscript.
